# Presence, Diversity, and Enrichment of Respiratory Reductive Dehalogenase and Non-respiratory Hydrolytic and Oxidative Dehalogenase Genes in Terrestrial Environments

**DOI:** 10.3389/fmicb.2019.01258

**Published:** 2019-06-07

**Authors:** Hanna R. Temme, Aaron Carlson, Paige J. Novak

**Affiliations:** Department of Civil, Environmental, and Geo- Engineering, University of Minnesota, Minneapolis, MN, United States

**Keywords:** reductive dehalogenation, reductive dehalogenase, haloalkane dehalogenase, chlorine cycle, chlorinated natural organic matter

## Abstract

Organohalide-respiring bacteria have been linked to the cycling and possible respiration of chlorinated natural organic matter (Cl-NOM) in uncontaminated soils and sediments. The importance of non-respiratory hydrolytic/oxidative dechlorination processes in the cycling of Cl-NOM in terrestrial soil and sediment, however, is still not understood. This research analyzes the dechlorination potential of terrestrial systems through analysis of the metagenomes of urban lake sediments and cultures enriched with Cl-NOM. Even with the variability in sample type and enrichment conditions, the potential to dechlorinate was universal, with reductive dehalogenase genes and hydrolytic or oxidative dehalogenase genes found in all samples analyzed. The reductive dehalogenase genes detected grouped taxonomically with those from organohalide-respiring bacteria with broad metabolic capabilities, as opposed to those that obligately respire organohalides. Furthermore, reductive dehalogenase genes and two haloacid dehalogenase genes increased in abundance when sediment was enriched with high concentrations of Cl-NOM. Our data suggests that both respiratory and non-respiratory dechlorination processes are important for Cl-NOM cycling, and that non-obligate organohalide-respiring bacteria are most likely involved in these processes.

## Introduction

Chlorinated organic chemicals, including polychlorinated biphenyls (PCBs), chlorinated ethenes, and chlorinated natural organic matter (Cl-NOM), are commonly found in soils and sediments (Blais et al., 1998; Major et al., 2002; Clarke et al., 2009; Aeppli et al., 2013; Rodenburg and Ralston, 2017). Although organochlorines can be present in high concentrations as a result of anthropogenic contamination events (e.g., Major et al., 2002), many sites are also impacted by low concentrations of chlorinated contaminants as a result of stormwater runoff and atmospheric deposition (Rodenburg et al., 2010; Curren et al., 2011; Gilbreath and McKee, 2015; McKee et al., 2017; Praipipat et al., 2017). Additionally, Cl-NOM is thought to be present at low concentrations in most soils and sediments, often as a function of chloride concentration (Johansson et al., 2003; Öberg and Sandén, 2005), made predominantly by fungi, bacteria, and plants, and in some cases via abiotic reactions (Asplund and Grimvall, 1991; Öberg and Grøn, 1998; Keppler et al., 2000; Keppler et al., 2002; Myneni, 2002; Bastviken et al., 2009; Vodyanitskii and Makarov, 2017). Cl-NOM is known to consist of over 5,000 different chemical structures (Gribble, 1992; Gribble, 2010), and can even include structures typical of contaminants, including trichloroethene (Abrahamsson et al., 1995), vinyl chloride (Keppler et al., 2002), tetrachloroethene (Weigold et al., 2015), and chlorinated phenols (Hoekstra et al., 1999).

Bacterial reductive dechlorination has been well studied (Adrian et al., 2007a; Krumins et al., 2009; Löffler et al., 2013; Jugder et al., 2015), with more recent attention given to the anaerobic bacterial dechlorination of Cl-NOM in uncontaminated terrestrial environments (Krzmarzick et al., 2012, 2014; Lim et al., 2018). Organohalide-respiring bacteria use reductive dechlorination to obtain energy for growth in a strictly anaerobic process by removing chlorine atoms from an organic backbone (reviewed by Adrian and Löffler, 2016). Interest in applying reductive dechlorination for the bioremediation of chlorinated contaminants has led to the purification and characterization of reductive dehalogenase proteins (reviewed by Jugder et al., 2015; Fincker and Spormann, 2017) and further identification of many more reductive dehalogenase genes (RDases) detected via DNA analysis (e.g., Adrian et al., 2007b; Krajmalnik-Brown et al., 2007; Tang et al., 2013). Putative RDase genes and organohalide-respiring bacteria have been found in uncontaminated environments, including marine sediment (e.g., Futagami et al., 2013; Kawai et al., 2014; Marshall et al., 2017), soil (e.g., Krzmarzick et al., 2012; Weigold et al., 2016), arctic soil (e.g., Zlamal et al., 2017), lake water (e.g., Biderre-Petit et al., 2016), and lake sediment (e.g., Krzmarzick et al., 2013), where they are thought to respire Cl-NOM. Nevertheless, it is unclear whether the amendment of Cl-NOM can enrich for RDases and whether the organisms involved in Cl-NOM dechlorination are more likely to be obligate or facultative organohalide respiring bacteria.

Other non-respiratory bacterial dechlorination processes also exist and use a variety of different hydrolytic or oxidative dehalogenase enzymes (reviewed in Fetzner, 1998; Ang et al., 2018). These processes result in the removal of chlorine atoms from an organic backbone with no energetic benefit to the organism, other than the liberation of organic carbon that can be used catabolically (Fetzner, 1998). The most common of these so-called non-reductive dehalogenase enzymes include haloalkane dehalogenases, 2-haloacid dehalogenases, 4-chlorobenzoyl-CoA dehalogenase, and fluoracetate dehalogenase, all of which function via hydrolytic dehalogenation (Ang et al., 2018), and a variety of mono- and dioxygenase enzymes that are specific to dechlorination, such as 2-halobenzoate 1,2-dioxygenase and chlorobenzoate dioxygenase (Fetzner, 1998). Haloalkane dehalogenases have been shown to be capable of dechlorinating contaminants, including hexachlorocyclohexane and dichloroethane (Sharma et al., 2006; Munro et al., 2017), and may also be capable of dechlorinating Cl-NOM, as suggested by Weigold et al. (2016). The majority of non-reductive dechlorination processes had been thought to be aerobic, but recently dehalogenase genes and hydrolytic dechlorination processes have also been found to be present or active in anaerobic and microaerophilic environments (Gossett, 2010; Bradley and Chapelle, 2011; Fullerton et al., 2014; Munro et al., 2017). It is currently unknown whether hydrolytic dehalogenases are common in anaerobic environments or whether they are generally active in terrestrial Cl-NOM dechlorination.

There is interest in the potential use of Cl-NOM to enrich organohalide respiring organisms and thereby stimulate contaminant dechlorination (e.g., Krzmarzick et al., 2012). Nevertheless, it is unknown whether reductive dechlorination is the dominant Cl-NOM dechlorination pathway in uncontaminated environments. Cl-NOM may instead be useful for stimulating hydrolytic or oxidative dechlorination. In addition, knowledge of the presence and abundance of non-respiratory hydrolytic/oxidative dechlorination genes versus reductive dechlorination genes in contaminated and uncontaminated environments could inform alternative remediation approaches, expanding the options available to dechlorinate contaminants under a variety of geochemical and environmental conditions. The goals of this research were therefore to determine whether uncontaminated versus contaminated sediments/soils would differentially dechlorinate Cl-NOM and mine the metagenomes of these systems to determine whether respiratory RDase genes or non-respiratory hydrolytic/oxidative dehalogenase genes were present, at what quantities, and whether they were enriched during Cl-NOM dechlorination. Metagenomes from additional differentially impacted urban lakes were also mined to evaluate whether similar types and numbers of dechlorinating genes were present in a variety of sediments, with the hypothesis that the more impacted lakes containing higher chloride concentrations would have a greater “natural enrichment” of RDase genes and hydrolytic/oxidative dehalogenase genes.

## Materials and Methods

### Sample Collection and Processing

**FIGURE 1:**
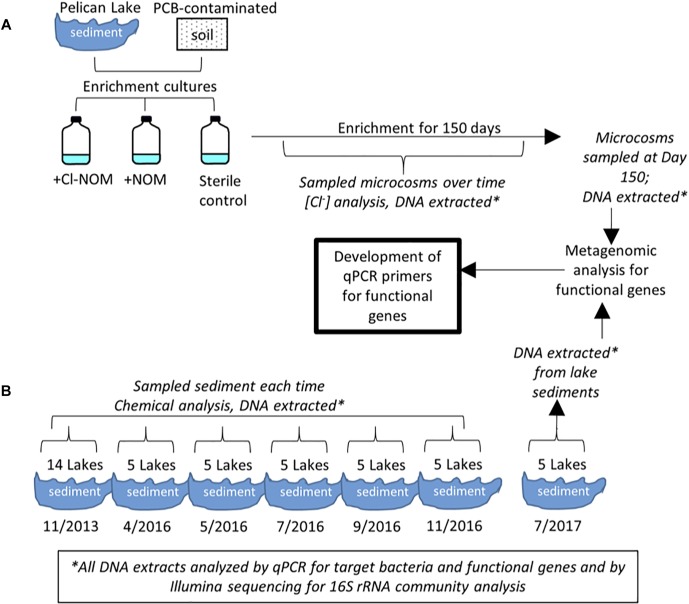
Schematic of the experimental set-up for **(A)** the sediment and soil enrichment cultures and **(B)** the lake samples.

Sample collection and use is shown schematically in Figure 1. Sediment samples from Pelican Lake, MN, United States (46° 36′ 42.7566″N, 94° 9′ 26.4024″W), were collected at a depth of 0.2–0.3 m below the water–sediment interface. Samples were placed in sterile air-tight containers with no headspace and were transferred to an anaerobic chamber (Coy) within 12 h of collection. A PCB-contaminated soil sample was supplied by a consulting company from a site undergoing remediation. This sample contained low concentrations of metals (<75 mg/kg) and moderate concentrations of total PCBs (approximately 40 mg/kg total PCBs). This material was shipped to the laboratory on ice and placed in an anaerobic chamber upon arrival before it was used to establish enrichment cultures (Figure 1).

The sediments of 14 lakes in the Minneapolis-St. Paul, MN, United States metropolitan area, impacted by different levels of chloride (Supplementary Table S1), were also sampled to study the presence of RDase or dehalogenase genes (Figure 1). These lakes have been monitored by the Minnesota Pollution Control Agency (MPCA) for 11–61 years (Wenck Associates, 2009), with 2007 being the last year of median chloride concentration data reported for all lakes. Initial sediment samples were taken in November 2013 at a depth of 0.2–0.3 m below the water–sediment interface. All samples were placed in air-tight containers with no headspace and were refrigerated at 4°C within 3 h of collection. Within 48 h of collection, the water and organic matter content, and the sediment pore water concentrations of chloride, bromide, nitrate, sulfate, and phosphate were determined as described below. DNA was extracted from each sediment sample using the MP Bio soil extraction kit, according to the manufacturer’s protocol, and stored at -20°C until analysis. Five of the 14 lakes (Bennett, Tanners, Square, Carver, and Turtle) were selected for additional sampling in 2016, on April 7, May 30, July 24, September 11, and November 20 (Figure 1). These were analyzed chemically and via qPCR as described below. Final samples of these five lake sediments were taken on July 22, 2017 and used for metagenomic sequencing (Figure 1). Sampling locations were the same for each sampling event and samples were always handled identically.

### Enrichment Cultures

The PCB-contaminated soil and Pelican Lake sediment microcosms were established in triplicate and enriched over 150 days (Figure 1). Enrichment cultures were constructed in 140-ml serum bottles containing 5 g of soil or sediment, 100 ml of reduced anaerobic mineral medium (RAMM) (Shelton and Tiedje, 1984) modified to have a low chloride content (see Supplementary Material), 1 mg/L of acetate as a carbon source, and 25 μl of either Cl-NOM or NOM (biological NOM control) in methanol. No external electron acceptors beyond Cl-NOM/NOM and HCO_3_^-^ were added to the enrichment cultures; nevertheless, other oxidized compounds that could serve as an electron acceptor, such as Fe^3+^, could have been present in the soil or sediment. The only differences between the sets of enrichment cultures was the addition of different soils/sediments and the presence of Cl-NOM vs. NOM. The Cl-NOM was synthesized from NOM extracted from Pelican Lake sediment using an automatic solvent extractor (ASE) with hexane:acetone (50:50); the NOM was then chlorinated using a chloroperoxidase enzyme, according to the protocol described by Krzmarzick et al. (2012). NOM-amended treatments contained the extracted NOM subject to the same processing as the Cl-NOM, except that the chloroperoxidase enzyme was not added (Krzmarzick et al., 2012). Autoclaved chemical negative controls were also prepared in triplicate and were identical to the Cl-NOM-amended microcosms, except that the soil or sediment had been autoclaved two times for 45 min before it was added to the serum bottles. Dechlorination was measured over time via chloride evolution, as described below. Student’s *t*-tests were used to determine if the quantity of chloride evolved was different between treatments. *P*-values < 0.05 were considered significant. Samples were taken for DNA analysis, extracted, and analyzed as described below.

### DNA Analysis

#### Metagenomic Sequencing

Metagenomic sequencing was completed on five of the unenriched urban lake sediments that were sampled July 2017 and on the Pelican Lake sediment and PCB-contaminated soil enrichment cultures after incubation with Cl-NOM or NOM for 150 days (Figure 1). DNA was extracted from the lake sediments after collection. For the enrichment cultures, 1 ml aliquots of triplicate microcosms from each treatment were sampled and the DNA extracted. The DNA from each triplicate microcosm after enrichment was pooled in equal volumes and sequenced for metagenomic analysis at the University of Minnesota Genomics Center (UMGC). Samples were pooled to increase the quantity of DNA for sequencing. The sediment samples were also sequenced at UMGC. Both sets of samples were sequenced on an Illumina HiSeq with paired end sequencing at 125 bp long; the enrichment culture samples were tagged, pooled, and sequenced on one lane and the sediment samples were tagged, pooled, and sequenced on a second lane. Nextera XT was used for library creation.

The sequencing data was analyzed at the Minnesota Supercomputing Institute (MSI). The Illumina adapters and primers were removed with the program Scythe. The reads were quality filtered and trimmed using the program Sickle using the default values for Sanger sequencing, as these are more stringent. FastQC was used to verify the read quality. Putative RDase and dehalogenase genes were then searched for in these unassembled reads using DIAMOND (Buchfink et al., 2015). Only *rdhA* genes were targeted in this work, but are referred to as RDase genes or enzymes, unless a specific version of the gene or enzyme is referred to. A database of RDase and dehalogenase enzymes was compiled from the curated Uniprot database and only contained enzyme sequences from bacteria that had been experimentally shown to be capable of dechlorination (Supplementary Table S2). This is a similar database to that used in a previous research study (Weigold et al., 2016), with the addition of enzymes verified since the cited work was published (2016). This database included RDases, haloalkane dehalogenases, S-2-haloacid dehalogenases, haloacetate dehalogenases, and dechlorination-specific mono- and dioxygenases. It did not include enzymes that are known to non-specifically dechlorinate, such as methane monooxygenase genes. DIAMOND was used to align the known RDase and dehalogenase enzymes to the unassembled reads using a cutoff of 60% protein identity and an e-value of 10^-9^ (Randle-Boggis et al., 2016). The relative abundance of each of the genes was calculated by taking the number of hits and dividing by the total number of quality-filtered reads (Weigold et al., 2016). Only one metagenome was analyzed per enrichment condition and per lake sediment, so it was not possible to perform statistics on these results. This resulted in what is referred to in the results section as “semi-quantitative” measurements of the gene abundance and language that is non-quantitative.

For reference, the abundance of the RDase and hydrolytic/oxidative dehalogenase genes in the unassembled reads were compared to genes that were part of the nitrogen cycle as a way to determine the relative abundance of different catabolic schemes. Consensus gene sequences of *nirK*, *nirS*, *amoA*, and *amoB* were obtained from the NCBI database and the metagenomic data was queried against these genes with DIAMOND, using the same search criteria used for RDase and hydrolytic/oxidative dehalogenase genes, as described above.

The metagenomic reads were also assembled for further analysis with the Iterative de Bruijn Graph Assembler (IDBA), with a minimum k value of 52, a maximum k value of 92, and a step of 8 (Peng et al., 2010). Information about the assembly quality is included in Supplementary Table S3. Longer fragments of RDases and hydrolytic or oxidative dehalogenases were also identified in these assemblies using DIAMOND. A less-stringent cutoff of 40% protein identity was used for this search, as the identified sequences were much less numerous and could therefore be manually checked against the NCBI database. The genes found in the assembled reads were then used to compare to previously identified hydrolytic/oxidative dehalogenase and RDase genes by aligning and trimming the sequences in Molecular Evolutionary Genetics Analysis (MEGA) software using Clustal, and creating phylogenetic trees using the maximum likelihood algorithm (Kumar et al., 2008).

#### qPCR Primer Development and Analysis

The putative RDases, haloalkane dehalogenase, and S-2-haloacid dehalogenase genes identified from the assembled contigs were used to create qPCR primers (Figure 1 and Table 1). The IDT PrimerQuest Tool was used to identify potential qPCR primers from the sequences. The identified primers were further analyzed via the IDT OligoAnalyzer tool and PrimerQuest tool for the potential to form primer dimers and secondary structures that would interfere with primer binding to the DNA target. Multiple primer sets were selected for each gene type and tested on a pooled sample of DNA that contained equal volume aliquots of the DNA from the five July 2016 lake sediment samples and the final samples taken from each of the enrichment cultures. Pooling the samples allowed for efficient testing of amplification with the multiple primer sets developed. The samples were run using the qPCR method described below, but with a gradient of annealing temperatures ranging from 53 to 62°C to determine optimal annealing temperatures for each primer set if amplification was achieved.

**Table 1:** qPCR primers developed from the metagenomes analyzed in this research.

Primer name	Sequence	Amplicon length (bp)	Annealing temp (°C)	Design target
TannersRDaseF TannersRDaseR	ACTATCGATCCGGAGAAGGT TCCTCCTCACTCCTCATATAGC	104	58.0	RDase gene detected in Tanners Lake
BennettRDaseF BennettRDaseR	CGAGGTCAACAGGCTTATC GGCACGGACTTCTCATTAC	87	58.0	RDase gene detected in Bennett Lake
PCBRDaseF PCBRDaseR	CCTGAACAGCTATGGGAATAC CAGCCGGTAATCAATACTCC	129	56.0	RDase gene detected in PCB-contaminated soil Cl-NOM enrichment
PelicanRDaseF PelicanRDaseR	GCTCGCCACCTTCATTACT GCCGTTCCGTCCCATTT	114	59.0	RDase gene detected in Pelican Lake Cl-NOM enrichment
TannersHaDhgF TannersHaDhgR	GAGAACCCTCATGGTCCTATCT CAGTTCTGCATCCAGTCCAC	157	56.0	Haloalkane dehalogenase gene detected in Tanners Lake
PCBHaDhg1F PCBHaDhg1R	CCATCAAATCGGGAGCTAAA CGTATGTGGATACAGGAAAGG	129	59.0	Haloalkane dehalogenase gene detected in PCB-contaminated soil Cl-NOM enrichment
PCBHaDhg2F PCBHaDhg2R	GGAACGCTTGATCTTGGAA CAAGGTAAGGGCGATGATATG	110	55.0	Haloalkane dehalogenase gene detected in PCB-contaminated soil Cl-NOM enrichment
CarverHaDhgF CarverHaDhgR	GTAGATGAGGGACCCAAGAA CACTCGATAACCTGCAACTG	114	56.0	Haloalkane dehalogenase gene detected in Carver Lake
PCB 2-haloacidDhgF PCB 2-haloacidDhgR	GTTTCGCATCCGGGTAAA GCTGACTTTCACGCTCAA	104	58.0	2-Haloacid dehalogenase gene detected in PCB-contaminated soil Cl-NOM enrichment
Pelican 2-haloacidDhgF Pelican 2-haloacidDhgR	CGATCCATGCCACATTCA GCGAACCTACGAACTGATT	122	56.0	2-Haloacid dehalogenase gene detected in Pelican Lake Cl-NOM enrichment


For assurance that the primer sets were targeting the correct novel dehalogenase or RDase gene target, the primer sets that resulted in amplification were further analyzed for specificity. To do this, the amplicons generated with the primer sets were sequenced on an Illumina Miseq at UMGC (300 bp paired end sequencing). The sequences were processed via the MSI. The reads were demultiplexed based on the primer sequences and then trimmed to 50 base pairs less than the expected amplicon length using Trimmomatic. The reads were further trimmed if needed, based on a Q-score of 30. The paired ends were interleaved and reads without matching paired reads were discarded. Using uclust, the reads were clustered at 100% similarity and clusters with less than three reads were disregarded to account for PCR and sequencing errors (Edgar, 2010). The sequences were compared to the NCBI database to determine whether the correct targeted sequences had been amplified by the primers. Only those primers that resulted in amplification of the targeted sequences in over 90% of the total reads were used in further sample analysis; these are shown in Table 1. Supplementary Table S4 shows the specificity of the primers used in this research, based on the Illumina sequencing.

The 16S rRNA genes of *Dehalococcoides mccartyi*, *Dehalococcoidia*, *Desulfitobacterium*, *Geobacter*, *Anaeromyxobacter*, *Desulfomonile*, *Dehalobium*, *Sulfurospirillum*, *Desulfovibrio*, *Dehalogenimonas*, and *Dehalobacter* were also quantified by qPCR in samples, in addition to determining the total bacteria 16S rRNA gene concentration (Muyzer et al., 1993; Fantroussi et al., 1997; Smits et al., 2004; Holmes et al., 2006; Amos et al., 2007; Himmelheber et al., 2009; Chen et al., 2014; Sutton et al., 2015; Wasmund et al., 2015). Because these primers were obtained from the literature, they were not further tested for specificity. No attempt was made to adjust gene copy numbers to number of organisms.

The qPCR mixture was the same for all of the primer sets and contained 1X SYBR green MasterMix (Bio-Rad Laboratories), 100 nM of each primer, 1 mg/L of BSA, and 1 μl of undiluted template in 15 μl reactions. The general qPCR cycle was an initial 95°C denaturation for 2 min followed by 40 cycles of 15 s 95°C denaturation and 30 s combined anneal/extension at the specific annealing temperature of each primer set (Table 1). A melt curve analysis was completed at the end of each run for quality control/assurance; a single peak that matched the standards in each case was consistently observed. The number of gene copies in each sample was determined with a standard curve of dilutions ranging from 10^8^ to 10^0^. These standards were purchased as gblocks from IDT, based on the sequence assembled from the metagenomes for the functional genes or the known 16S rRNA gene sequences available in the NCBI database. The standards were all 500 bp long with the primer target in the middle of the gene fragment to allow for improved primer binding. DNA sequences for the RDase and hydrolytic dehalogenase standards are included in the Supplementary Material. The general 16S rRNA gene standards for quantifying total bacteria were prepared by ligating the 16S rRNA gene from *Escherichia coli* into pGEM-T Easy vectors (Promega) according to the manufacturer instructions. This was transformed into *E. coli* JM109. Plasmids were purified using a MiniPrep Kit (Qiagen) and quantified by UMGC using the PicoGreen method. The limit of quantification for the developed primer sets for the functional genes was 10 copies/μl. The limit of quantification for the generic 16S primer set was 10^3^ copies/μl. The limit of quantification for all other primer sets was 10^2^ copies/μl.

For each putative RDase and hydrolytic dehalogenase gene measured, Spearman’s rank and point biserial correlations were used to determine if there was a statistical relationship between the log of a given gene concentration and either the chloride concentration or the presence of a chloride impairment, respectively. For the point biserial correlation, the binary MPCA classification of “impaired” or “not impaired” was used. The MPCA considers a water body impaired for chloride if the chloride concentrations exceeded 230 mg/L for at least two samples in a 3-year period (Wenck Associates, 2009).

### Analytical Methods

The water content of the collected sediments was measured by weighing the samples before and after they were dried at 105°C. The organic matter loss on ignition was measured by gravimetrically comparing the dry samples before and after burning at 550°C for 4 h. This was used to normalize the qPCR results.

Ion chromatography (IC) was used to measure bromide, chloride, nitrate, phosphate, and sulfate in the pore water of the urban lake sediments. IC was also used to quantify chloride release in the Cl-NOM and NOM enrichment cultures. The same method was used for both measurements. Samples were centrifuged and the supernatant was injected into a Metrohm 930 Compact IC Flex with a Metrosep A sup 5 anion separation column. The flow rate of the eluent of carbonate buffer (3.2 mM Na_2_CO_3_ and 1.0 mM NaHCO_3_) was 0.7 ml/min. The detection limits for bromide, nitrate, phosphate, and sulfate were 0.005 mg/L while the detection limit for chloride was 0.01 mg/L.

## Results

### Dechlorination and Gene Detection With Cl-NOM Enrichment

**FIGURE 2:**
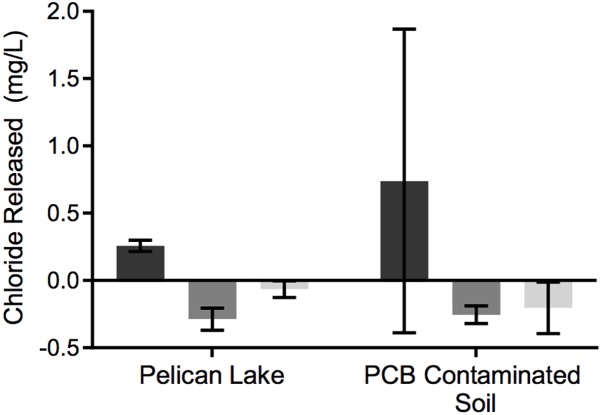
Chloride released during enrichment with Cl-NOM. The chloride released was assumed to be a result of Cl-NOM dechlorination. Dark gray bars are the Cl-NOM enrichments, medium gray are the NOM enrichments, and light gray are the sterile negative controls. Error bars represent the standard deviation of triplicate enrichment cultures.

The uncontaminated Pelican Lake sediment and the PCB-contaminated soil differentially dechlorinated Cl-NOM, with Cl-NOM consistently dechlorinated more in the uncontaminated Pelican Lake sediment (Figure 2). Pelican Lake sediment cultures also released significantly more chloride in the Cl-NOM-amended versus NOM-amended enrichment cultures (*P* = 0.001), demonstrating that the amended Cl-NOM did stimulate dechlorination in these cultures. Although there was some Cl-NOM dechlorination in the PCB-contaminated soil enrichment cultures, it was inconsistent and chloride release was statistically similar in the Cl-NOM-amended and NOM-amended treatments (*P* = 0.13, Figure 2). One of the triplicate enrichment cultures did show a large increase in chloride concentration (2.0 mg/L) over the 150 days, but this was not observed in the other two enrichment cultures (0.16 and 0.02 mg/L chloride released). These results show that prior exposure to a chlorinated pollutant did not enhance the ability of the PCB-contaminated soil to dechlorinate Cl-NOM, but, as the precursor NOM used to generate the Cl-NOM was derived from Pelican Lake, prior exposure to NOM, some of which was likely naturally chlorinated, did enhance Cl-NOM dechlorination.

When analyzed semi-quantitatively using the metagenome data, putative RDase genes and hydrolytic and oxidative dehalogenase genes were detected in the enrichment cultures, regardless of previous exposure to Cl-NOM or PCBs or enrichment on NOM or Cl-NOM (Table 2), indicating substantial potential for dechlorination. In fact, these genes were present at concentrations similar to genes commonly used for nitrogen cycling (Table 3), further suggesting that this ability may be common. The hydrolytic/oxidative dehalogenase genes were typically more abundant, with haloalkane dehalogenase and the 2-haloacid dehalogenase genes detected most frequently (Table 2). In the Pelican Lake sediment enrichment cultures, the metagenomes taken after 150 days of enrichment showed a higher frequency of RDase genes in the Cl-NOM enrichment cultures compared to the NOM-amended controls, with between 2 and 84 genes per 10 million reads identified in the various treatments (Table 2). In the metagenomes of the PCB-contaminated soil microcosms, however, the frequency of RDase gene detection was similar between the Cl-NOM and NOM enrichment cultures (Table 2). This mirrored the chloride release patterns (Figure 2) and suggested that the presence (or amendment) of high concentrations of Cl-NOM could enrich for RDase genes in some, but not all, terrestrial soils/sediments. With respect to the abundant and diverse hydrolytic and oxidative dehalogenase genes identified in the metagenomes, detection frequency did not mirror the observed dechlorination patterns, with similar abundance profiles between the Cl-NOM- and NOM-amended Pelican Lake sediment and PCB-contaminated soil treatments (Table 2). This is perhaps expected, as between 29 and 110 non-reductive dehalogenase genes per 10 million reads were identified in the different cultures after 150 days of enrichment, indicating that the genes were common and might have a large number of functions not tied to Cl-NOM dechlorination.

**Table 2:** Frequency of RDase or hydrolytic/oxidative dehalogenase genes detected in the metagenomes generated from the Cl-NOM and NOM enrichments.

	Pelican ClNOM	Pelican NOM	PCB ClNOM	PCB NOM
Reductive dehalogenase genes	84	2	16	18
Haloalkane dehalogenase genes	20	22	49	85
2-Haloacid dehalogenase genes	1	1	17	10
Haloacetate dehalogenase genes	2	2	1	3
Di- and monooxygenase dehalogenase genes	8	4	20	8
Other dehalogenase genes	5	0.7	6	4


*The numbers shown are gene copies per 10 million reads. A full list of the hydrolytic/oxidative dehalogenase genes found is included in the Supplementary Material.*

**Table 3:** Frequency of RDase or hydrolytic/oxidative dehalogenase genes detected in the metagenomes generated from the lake sediment.

	Square	Bennett	Turtle	Tanners	Carver
Reductive dehalogenase genes	23	17	11	30	25
Haloalkane dehalogenase genes	83	53	79	64	59
2-Haloacid dehalogenase genes	12	8	20	4	7
Haloacetate dehalogenase genes	23	12	23	9	13
Di- and monooxygenase dehalogenase genes	11	13	17	9	12
Other dehalogenase genes	8	10	5	6	9
*nirK*	283	47	773	244	276
*nirS*	13	9	41	3	7
*amoA*	23	6	10	7	6
*amoB*	26	3	15	0	8
Median chloride	35.3	160	150	146	151.3
Watershed road density (percent)	>18	23	<18	29	25


*The numbers shown are gene copies per 10 million reads. A full list of the hydrolytic/oxidative dehalogenase genes found is included in the Supplementary Material.*

**FIGURE 3:**
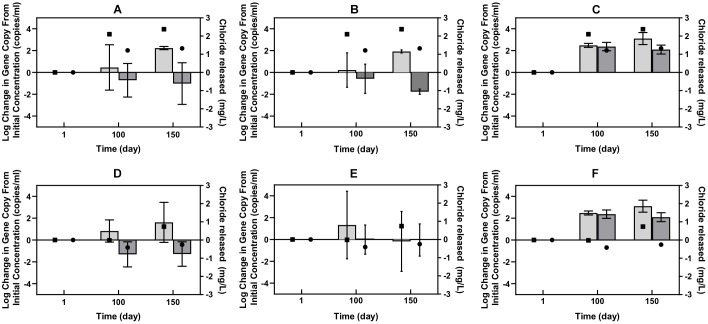
Log change in the number of gene copies of three specific hydrolytic dehalogenase genes from the levels present at time zero. The figure panels show Pelican Lake enrichment cultures **(A–C)** and PCB contaminated soil enrichments **(D–F)**. Shown are results using the PCB 2-haloacidDhg **(A,D)**, the Pelican 2-haloacidDhg **(B,E)**, and PCBHaDhg1 **(C,F)** primer sets. The light gray bars show the data for the Cl-NOM-amended treatments and the dark gray bars show that from the NOM-amended treatments. The error bars represent standard deviations of triplicates. The right *y*-axis shows the average chloride released at each time point for the Cl-NOM enrichments (square) and NOM enrichments (circle).

The qPCR results corroborated the metagenomic results to some extent, but enabled a more quantitative and specific look at several of the genes associated with Cl-NOM dechlorination. Successful development of a qPCR method was possible with only one of the many RDase genes identified in the metagenome, which did not show enrichment during Cl-NOM dechlorination (Supplementary Figure S1). The qPCR data of the hydrolytic dehalogenase genes, however, did indicate a link between Cl-NOM dechlorination and the enrichment of specific genes in both the Pelican Lake sediment treatments and in the Cl-NOM-amended single PCB-contaminated soil enrichment in which substantial chloride evolution was observed (Figure 3). The quantities of two different 2-haloacid dehalogenase genes increased significantly more in the enrichment cultures in which Cl-NOM was dechlorinated compared to those amended with NOM (*P* = 0.002, *P* = 0.04, Figure 3) and log increases in both of the hydrolytic dehalogenase genes of 2.9 genes/ml and 3.6 genes/ml were observed in the PCB-contaminated soil enrichment in which dechlorination was observed. One haloalkane dehalogenase gene also increased in number in the Pelican Lake sediment enrichment cultures in which Cl-NOM was dechlorinated (Figure 3), but this increase was not significant at the 95% confidence level when compared to the increase of the same gene observed in the NOM-amended treatments (*P* = 0.09). Other than the single Cl-NOM-amended treatment that demonstrated substantial dechlorination, all of the genes monitored by qPCR decreased in the Cl-NOM- and NOM-amended enrichment cultures containing the PCB contaminated soil (Figure 3 and Supplementary Figure S1).

Taken together, the metagenomic and qPCR data suggest that multiple RDase genes and abundant hydrolytic and oxidative dehalogenase genes were present in these two sediments/soils and that Cl-NOM amendment stimulated Cl-NOM dechlorination, which, while it may have been linked to the activity of RDase genes (Table 2), appeared to be clearly linked to the enrichment of two haloacid dehalogenase genes, and possibly to enrichment of a haloalkane dehalogenase gene. Nevertheless, the focus on DNA in this research, as opposed to mRNA transcripts or functional enzymes (e.g., Tang et al., 2013) does limit the work and makes it impossible to definitively link particular genes to Cl-NOM dechlorination.

### RDase and Hydrolytic Dehalogenase Gene Presence and Abundance in Urban Lakes

**FIGURE 4:**
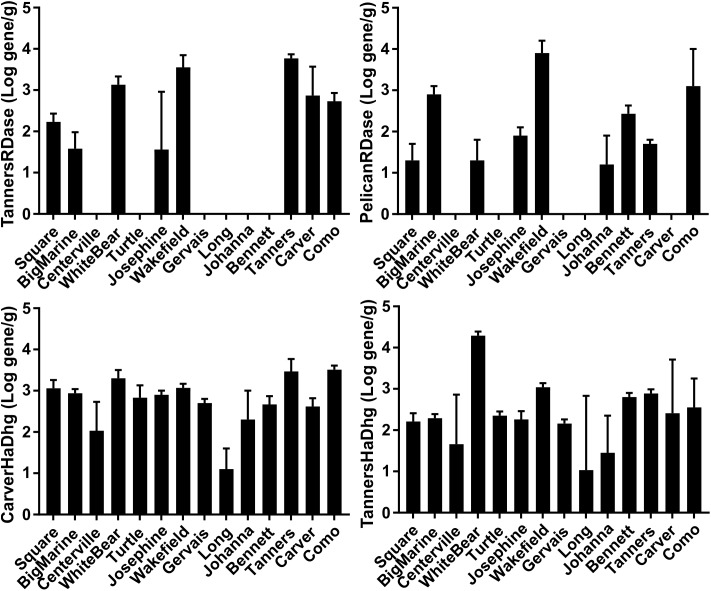
qPCR results of selected genes from lakes with varying urban impact: TannersRDase, PelicanRDase, CarverHaDhg, and TannersHaDhg. The lakes are ordered according to increasing median salt concentration (Supplementary Table S1) as a measure of urban impact, with Square Lake as the least impacted and Como Lake as the most impacted. All measurements are shown in units of log gene copy per gram sediment.

Urban lakes were sampled across a range of urban impact with the hypothesis that the presence of chloride in more heavily impacted lakes would result in a “natural enrichment” of RDase and/or hydrolytic/oxidative dehalogenase genes. The metagenome and qPCR results indicated the presence of multiple putative RDase (11–30 per 10 million reads) and hydrolytic and oxidative dehalogenase genes (92–144 per 10 million reads), again, at levels similar to that observed for those genes commonly used for nitrogen cycling (Table 3). Some of the RDase genes and hydrolytic/oxidative dehalogenase genes were common among multiple lake sediments and some appeared to be sediment/soil-specific (Figure 4, Table 3, and Supplementary Tables S5, S6). The quantities of the different putative dechlorinating genes also ranged over several orders of magnitude in the different sediments (Supplementary Tables S5, S6). For example, the RDase gene originally found in the Tanners Lake sediment metagenome was present above the limit of quantification, and ranging in quantity over two orders of magnitude, in 8 of the 14 lakes (Supplementary Table S5). An RDase found in the Pelican Lake sediment enrichment culture metagenome was detected in 9 of the 14 lake sediments, ranging in quantity over nearly three orders of magnitude (Supplementary Table S5). Similarly, two of the haloalkane dehalogenase genes that were originally sequenced in the lake sediment metagenomes were present in all 14 lakes and one of the haloalkane dehalogenases originally sequenced in the Cl-NOM enrichment cultures established with the PCB-contaminated soil was also found in 5 out of the 14 urban lakes (Figure 4 and Supplementary Table S6). Nevertheless, despite the large number of RDase (11–30 per 10 million reads) and hydrolytic and oxidative dehalogenase genes (92–144 per 10 million reads) identified, neither the numbers of the genes detected via the metagenome analysis nor the spatial or temporal dynamics of the genes (Supplementary Table S7), as determined by qPCR, correlated to urban impact (Supplementary Table S8), demonstrating a lack of the hypothesized “natural enrichment” for dechlorination ability.

### Taxonomic Relationships of RDase and Hydrolytic Dehalogenase Genes

**FIGURE 5:**
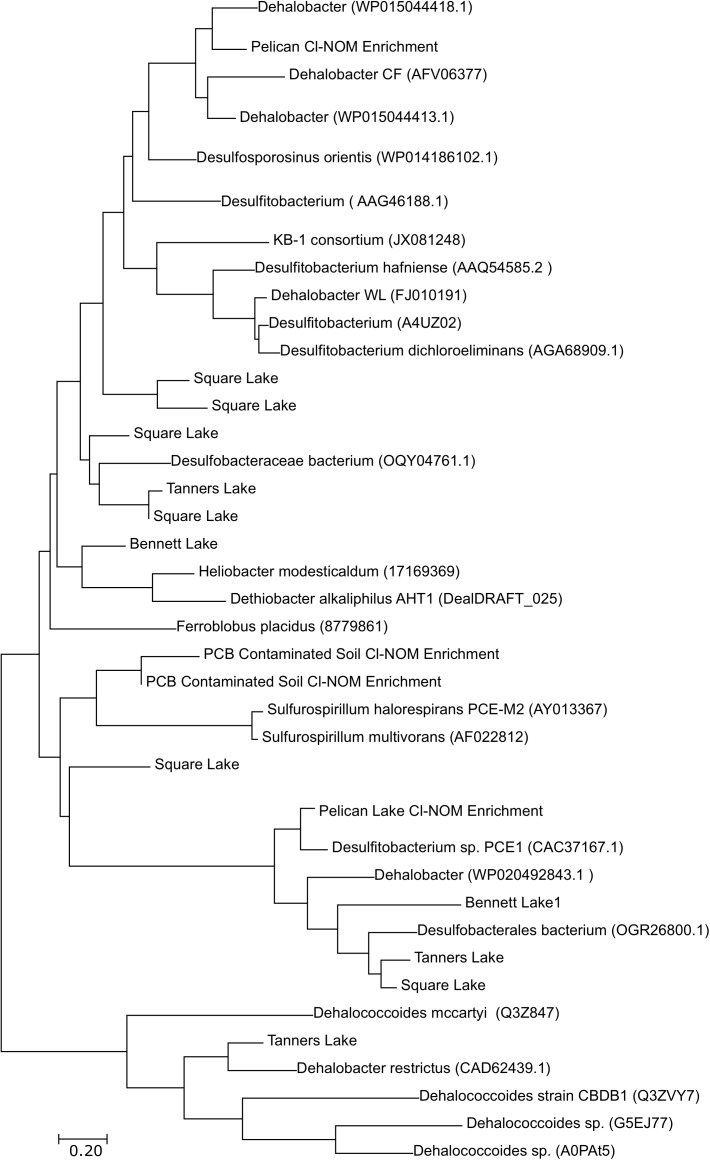
Taxonomic relationships of the putative RDase genes sequenced in the urban lakes and the Cl-NOM enrichment cultures. The NCBI, JGI, or UniProt ID numbers are given in parentheses. The figure does not provide a comprehensive list of previously identified RDase genes and includes disproportionally more of the RDase genes found in the *Firmicutes* phylum. A haloalkane dehalogenase gene was used as an outgroup. The tree was constructed using the maximum likelihood algorithm in Mega5. Sequences were aligned using Clustal.

Although *Dehalococcoides*, *Geobacter*, *Desulfomonile*, *Anaeromyxobacter*, *Desulfovibrio*, and *Dehalogenimonas* spp. were all present in numbers generally greater than 10^3^ gene copies/g wet sediment (Supplementary Table S9), taxonomically, the RDase genes detected via the metagenomic analysis in both the enrichment cultures and lake sediments were most similar to RDase genes belonging to *Desulfitobacter*, *Dehalobacter*, *Sulfurospirillum* spp. and other bacteria found in the *Firmicutes* and *Proteobacteria* phyla (Figure 5). The only RDase gene similar to those found in *Dehalococcoides mccartyi* was identified in Tanners Lake (Figure 5). This is interesting because qPCR results showed higher numbers of 16S rRNA genes from *Dehalococcoides* and *Dehalogenimonas* spp. compared to the other putative organohalide-respiring bacteria quantified (Supplementary Table S9). These results suggest that the RDase genes detected via the metagenomic analysis may reside in putative organohalide respiring bacteria belonging to the *Firmicutes* and *Proteobacteria* phyla that were not quantified by qPCR. Previous work has suggested that there may be organohalide-respiring bacteria in both the *Firmicutes* and *Proteobacteria* phyla that have yet to be identified (Chang et al., 2000; Ahn et al., 2009; Krzmarzick et al., 2014). Known organohalide-respiring bacteria in the *Proteobacteria* and *Firmicutes* phyla tend to have metabolic capabilities beyond reductive dechlorination (Sun et al., 2001; Sanford et al., 2002; Luijten et al., 2003; Sung et al., 2006), with the exception of *Dehalobacter*, the known isolate of which is an obligate organohalide respiring bacterium. This metabolic flexibility could allow them to survive primarily on non-chlorinated electron acceptors, while continuing to dechlorinate the low concentrations of organochlorines that would be expected in urban-impacted lake sediments. Indeed, this could explain the lack of a correlation between urban impact and the number or dynamics of RDase genes, as the organisms that harbor these genes in relatively uncontaminated environments may be metabolic “generalists,” consuming a mixture of low-level electron acceptors rather than obligate organohalide respiring “specialists.” In contrast, the specific enrichment of cultures on high concentrations of Cl-NOM would be more likely to result in enrichment of putative RDases, as observed. Some of the RDase genes identified in this study did not cluster with previously identified RDase genes. This is unsurprising, as limited work has been performed to understand the diversity of RDase genes in uncontaminated sediments and it is therefore likely that novel RDase genes would be present in these environments. Finally, the RDase genes identified did not group taxonomically as a result of urbanization or previous organohalide exposure, with two RDase genes identified in Square Lake sediment (least impacted) clustering near two RDase genes identified from Tanners Lake sediment (most impacted).

The haloalkane dehalogenase genes identified from the metagenomes were also analyzed to determine their taxonomic relationships to known genes. Haloalkane dehalogenase genes have been found to separate into distinct groups (Chovancová et al., 2007); in this analysis, the genes identified in the lake sediments were in a different group than those identified in the Cl-NOM enrichment cultures (Supplementary Figure S2). The haloalkane dehalogenase genes identified in the PCB-contaminated soil and Pelican Lake sediment, however, were similar, which may have been a result of the similar enrichment conditions. Within the lake sediment samples, the haloalkane dehalogenase genes in Tanners Lake and Square Lake sediments were the most similar, while those identified in Carver and Turtle Lakes were also more similar to each other. No haloalkane dehalogenase genes were identified in the assembled contigs from Bennett Lake sediment. Because dehalogenase genes are broadly distributed phylogenetically (Janssen et al., 2005; Chovancová et al., 2007), it is impossible to speculate on the specific types of organisms that harbor these genes and their inherent metabolic capabilities.

## Discussion

The presence of putative RDase and dehalogenase genes in multiple urban lake sediments and cultures enriched on NOM and Cl-NOM demonstrates that the potential to dechlorinate is widespread. In fact, each sample analyzed contained a variety of genes encoding for enzymes thought to be capable of dehalogenation. These genes were present at concentrations similar to genes commonly used for nitrogen cycling (Table 3), demonstrating that not only is this ability widespread, it may also be common.

Some putative RDase and dehalogenase genes were common to multiple lake sediments and enrichment cultures, while other genes were specific to a single location or a single culture. Previously published work found that the RDase genes or bacteria known to contain these enzymes were widely present at contaminated sites (Hendrickson et al., 2002; Müller et al., 2004; Mattes et al., 2017). RDase genes have also been identified in uncontaminated marine environments where concentrations of Cl-NOM are higher (Kawai et al., 2014) and in select terrestrial environments (Biderre-Petit et al., 2016; Weigold et al., 2016). Bacteria taxonomically similar to known organohalide-respiring bacteria have also been previously detected in uncontaminated marine and terrestrial environments (Futagami et al., 2013; Krzmarzick et al., 2013; Biderre-Petit et al., 2016), and in one case their growth was linked to the dechlorination of Cl-NOM (Krzmarzick et al., 2012). The research presented herein furthers our knowledge regarding the dechlorination potential of uncontaminated, or relatively uncontaminated, terrestrial environments, suggesting, based on the presence of functional genes, that this potential is widespread.

The presence and likely involvement of non-respiratory dehalogenase genes in Cl-NOM dechlorination is particularly interesting. Indeed, both RDase and dehalogenase genes increased in detection frequency during Cl-NOM dechlorination (Table 2 and Figure 4), suggesting enrichment of these genes during dechlorination. Dehalogenase enzymes have been found to be capable of dechlorinating contaminants (Sharma et al., 2006; Arbeli and Fuentes, 2010; Munro et al., 2016), and a recent study showed that haloalkane dehalogenase genes were detected at higher concentrations than RDase genes in a dichloroethane-contaminated plume showing signs of active dechlorination (Munro et al., 2017). Non-reductive dehalogenase genes have primarily been studied in aerobic environments, but their presence at higher frequencies and similar individual concentrations to RDase genes in our samples and in the recent literature provides evidence that hydrolytic and/or oxidative dechlorination is active in anaerobic environments. These results also suggest that hydrolytic, and possibly oxidative dehalogenase enzymes might be as important as RDase enzymes for Cl-NOM (and perhaps contaminant) dechlorination in anaerobic environments (Munro et al., 2017).

Further research on non-respiratory dechlorination may be of particular use when considering alternative strategies to stimulate contaminant dechlorination. Perhaps stimulation of non-obligate organohalide-respiring bacteria in the *Firmicutes* and *Proteobacteria* phyla and of bacteria containing hydrolytic and/or oxidative dehalogenase genes through the supply of nutrients (nitrogen, phosphorus, etc.) but limited quantities of carbon may allow for dechlorination at sites that do not appear to respond to the stimulation of organohalide respiring bacteria. It is possible that a more expansive view of bioremediation, including efforts at stimulating both organohalide-respiring bacteria and non-respiratory dechlorinating bacteria, could be more effective in the clean-up of chlorinated contaminants, particularly those present at lower concentrations, where RDase gene quantities and expression may be lower.

## Contribution to the Field

Genes encoding for enzymes capable of dechlorination have been previously found at sites with no known organochlorine contamination; those studies focused on a single location. This work surveyed multiple lakes with varying levels of urban impact and found the potential to dechlorinate was present in every site analyzed. This suggests that dechlorination could be a common metabolic capability in terrestrial systems. This work also highlights the importance of non-respiratory dehalogenase genes and chlorinated natural organic matter (Cl-NOM) cycling. In bioremediation, respiratory reductive dehalogenase genes are typically the focus for stimulation; nevertheless, the presence of non-respiratory hydrolytic and/or oxidative dehalogenase genes at high frequencies in the samples studied herein shows that these genes may also play an important role in the global chlorine cycle and may be utilized in bioremediation, especially at low organochlorine concentrations.

## Data Availability

The datasets generated for this study can be found in the NCBI database under BioProject PRJNA483483 (http://www.ncbi.nlm.nih.gov/bioproject/483483).

## Author Contributions

HT was responsible for the majority of the experimental design, data collection, analysis, and writing of the manuscript. PN aided with the experimental design, data analysis, and editing of the manuscript. AC was responsible for significant portions of the chemical data collection and analysis of that data.

## Conflict of Interest Statement

The authors declare that the research was conducted in the absence of any commercial or financial relationships that could be construed as a potential conflict of interest.
