# Supplementary material for: Presence, Diversity, and Enrichment of Respiratory Reductive Dehalogenase and Non-respiratory Hydrolytic and Oxidative Dehalogenase Genes in Terrestrial Environments
Source: Front Microbiol. 2019 Jun 7;10:1258. doi: 10.3389/fmicb.2019.01258 (PMC6567934; doi:10.3389/fmicb.2019.01258)
Supplement: Supplementary file 1 [file Data_Sheet_1.pdf]

Supplementary Material

1 Methodological details

Low Chloride RAMM Medium

The low chloride RAMM contained 0.27 g KH_2PO_4, 0.35 g K_2HPO_4, 0.97 g NH_4Br, 51 mg $CaBr_2$, 90.5 mg MgBr, 36.3 mg $FeBr_3$, and 10 ml of the reduced chloride mineral solution mixed into 1 L of ultrapure water (MilliQ, Millipore). Resazurin was added as an indicator. If needed, the media was then pH adjusted to approximately 7.0. The mineral solution contained 72.4 mg $MnBr_2$•4 H_20, 5 mg H_3BO_3, 3.2 mg CuBr, 1 mg $NaMoO_4$•$2H_2O$, 80 mg $CoBr_2$•H_2O, 4.5 mg $NiBr_2$, and 5 mg Na_2SeO_3 in 1 L of ultrapure water (MilliQ, Millipore). The chloride concentration of the prepared microcosms was approximately 1.5 mg/L.

Table S1. Lakes sampled in November 2013. The median chloride concentration, chloride impairment, and watershed road density were based on data collected by the Minnesota Pollution Control Agency (Wenck Associates 2009).

**Lake**	**Sampling Coordinates**	**Median 2007 chloride concentration (mg/L)**	**Chloride Impairment[a]**	**Watershed road density %**
Big Marine	45.206892, -92.872495	11	no	<18
Tanners	44.953605, -92.978937	161.7	yes	29
White Bear	45.092946, -92.995624	35.3	no	<18
Gervais	45.014649, -93.075463	150	no, high risk[b]	29
Carver	44.904675, -92.979677	190	yes	25
Bennett	45.019063, -93.141042	160	no, high risk	23
Square	45.154908, -92.795003	7	no	<18
Long	45.076739, -93.200628	151.3	yes	33
Turtle	45.091829, -93.131323	40	no	<18
Wakefield	44.994585, -93.034181	146	no, high risk	26
Johanna	45.047598, -93.173953	152.5	no, high risk	36
Centerville	45.160146, -93.081317	26	no, high risk	<18
Como	44.982706, -93.140447	192.5	yes	32
Josephine	45.034476, -93.149392	65	no	21

[a] Lakes were considered impaired for chloride if they had chloride concentrations above 230 mg/L during low flow conditions.

[b] High risk lakes were lakes with higher chloride concentration that were also increasing in chloride concentration over the period of analysis.

Table S2. List of RDase and hydrolytic/oxidative dehalogenase genes found in the metagenomes from the Uniprot database.

Uniprot Number	Entry Name	Protein name	Organism
P42535	PCPB_SPHCR	Pentachlorophenol 4-monooxygenase	*Sphingobium chlorophenolicum*
Q8KN28	TFDB_DELAC	2,4-dichlorophenol 6-monooxygenase	*Delftia acidovorans*
P27138	TFDB_CUPPJ	2,4-dichlorophenol 6-monooxygenase	*Cupriavidus pinatubonensis*
Q51603	CBDC_BURCE	2-halobenzoate 1,2-dioxygenase	*Burkholderia cepacia*
Q51601	CBDA_BURCE	2-halobenzoate 1,2-dioxygenase	*Burkholderia cepacia*
Q51602	CBDB_BURCE	2-halobenzoate 1,2-dioxygenase	*Burkholderia cepacia*
Q44257	CBAB_COMTE	3-chlorobenzoate-3,4-dioxygenase reductase	*Comamonas testosteroni*
Q44256	CBAA_COMTE	3-chlorobenzoate-3,4-dioxygenase oxygenase	*Comamonas testosteroni*
Q6EM25	Q6EM25_9PSED	Chlorocatechol 2,3-dioxygenase	*Pseudomonas* sp. MG61
Q6EM26	Q6EM26_9BACT	Chlorocatechol 2,3-dioxygenase	*enrichment culture*
Q6EM23	Q6EM23_PSEFL	Chlorocatechol 2,3-dioxygenase	*Pseudomonas fluorescens*
O85078	CBAD1_ARTSP	4-chlorobenzoyl coenzyme A dehalogenase	*Arthrobacter*
P12015	CYMO_ACISP	Cyclohexanone 1,2-monooxygenase	*Acinetobacter*
Q53464	HAD_PSEUY	(S)-2-haloacid dehalogenase	*Pseudomonas*
Q60099	HAD_XANAU	(S)-2-haloacid dehalogenase	*Xanthobacter autotrophicus*

Q52087	HADL_PSEPU	(S)-2-haloacid dehalogenase	*Pseudomonas putida*
Q59728	HAD9_PSEPU	(S)-2-haloacid dehalogenase	*Pseudomonas putida*
P24069	HAD1_PSEUC	(S)-2-haloacid dehalogenase	*Pseudomonas*
Q59666	HAD_PSEFL	(S)-2-haloacid dehalogenase	*Pseudomonas fluorescense*
Q51645	HAD4_BURCE	(S)-2-haloacid dehalogenase	*Burkholderia cepacia*
P22643	DHLA_XANAU	Haloalkane dehalogenase	*Xanthobacter autotrophicus*
P51698	LINB_SPHPI	Haloalkane dehalogenase	*Sphingomonas paucimobilis*
P0A3G3	DHAA_RHOSO	Haloalkane dehalogenase	*Rhodococcus* sp.
P59337	DHAA_BRADU	Haloalkane dehalogenase	*Bradyrhizobium diazoefficiens*
Q50642	DHAA_MYCTU	Haloalkane dehalogenase	*Mycobacterium tuberculosis*
P64301	DHMA1_MYCTU	Haloalkane dehalogenase	*Mycobacterium tuberculosis*
P59336	DHAA_RHOSD	Haloalkane dehalogenase	*Rhodococcus*
Q938B4	DHAA_MYCS2	Haloalkane dehalogenase	*Mycobacterium smegmatis*
P64303	DHMA2_MYCTU	Haloalkane dehalogenase	*Mycobacterium tuberculosis*
Q9ZER0	DHAA_MYCSX	Haloalkane dehalogenase	*Mycobacterium*
A1KLS7	DHAA_MYCBP	Haloalkane dehalogenase	*Mycobacterium bovis*
Q98C03	DHAA_RHILO	Haloalkane dehalogenase	*Rhizobium loti*
Q9XB14	DHAA_MYCBO	Haloalkane dehalogenase	*Mycobacterium bovis*
B8H3S9	DHMA_CAUCN	Haloalkane dehalogenase	*Caulobacter crescentus*
Q9A919	DHMA_CAUCR	Haloalkane dehalogenase	*Caulobacter crescentus*

B2HJU9	DHMA_MYCMM	Haloalkane dehalogenase	*Mycobacterium marinum*
Q73Y99	DHMA_MYCPA	Haloalkane dehalogenase	*Mycobacterium paratuberculosis*
Q8U671	DHAA_AGRT5	Haloalkane dehalogenase	*Agrobacterium tumefaciens*
B4RF90	DHMA_PHEZH	Haloalkane dehalogenase	*Phenylobacterium zucineum*
B0SY51	DHMA_CAUSK	Haloalkane dehalogenase	*Caulobacter*
Q1QBB9	DHMA_PSYCK	Haloalkane dehalogenase	*Psychrobacter cryohallolentis*
E5G1F0	E5G1F0_9PROT	PceA protein	*Sulfurospirillum*
E5G1E9	E5G1E9_9PROT	PceA protein	*Sulfurospirillum*
Q939S2	Q939S2_9PROT	Putative tetrachloroethen reductive dehalogenase	
Q8L172	Q8L172_DESHY	PCE dehalogenase	*Desulfitobacterium hafniense*
Q8GJ27	Q8GJ27_9FIRM	Tetrachloroethene reductive dehalogenase	*Dehalobacter restrictus*
A4UZ02	A4UZ02_9FIRM	Putative PCE dehalogenase	*Desulfitobacterium*
B5LNP4	B5LNP4_9FIRM	Tetrachloroethene reductive dehalogenase	*Desulfitobacterium*
B5LNQ0	B5LNQ0_9FIRM	Tetrachloroethene reductive dehalogenase	*Desulfitobacterium*
B5LNP8	B5LNP8_9FIRM	Tetrachloroethene reductive dehalogenase	*Desulfitobacterium*
B5LNQ1	B5LNQ1_9FIRM	Tetrachloroethene reductive dehalogenase	*Desulfitobacterium*
O68252	O68252_DEHMU	Tetrachloroethene reductive dehalogenase	*Sulfurospirillum multivorans*

Q3ZVY7	Q3ZVY7_DEHSC	Putative reductive dehalogenase	*Dehalococcoides*
A0PAT0	A0PAT0_9CHLR	Trichloroethene reductive dehalogenase	uncultured *Dehalococcoides*
Q3ZAB8	Q3ZAB8_DEHE1	Trichloroethene reductive dehalogenase	*Dehalococcoides mccartyi*
Q9KIP5	Q9KIP5_9CHLR	Trichloroethene reductive dehalogenase	*Dehalococcoides mccartyi*
Q3Z847	Q3Z847_DEHE1	Putative reductive dehalogenase	*Dehalococcoides mccartyi*
Q3Z6A6	Q3Z6A6_DEHE1	Putative reductive dehalogenase	*Dehalococcoides mccartyi*
Q3Z6D2	Q3Z6D2_DEHE1	Putative reductive dehalogenase	*Dehalococcoides mccartyi*
Q3Z9P5	Q3Z9P5_DEHE1	Putative reductive dehalogenase	*Dehalococcoides mccartyi*
Q3Z9P0	Q3Z9P0_DEHE1	Putative reductive dehalogenase	*Dehalococcoides mccartyi*
Q3ZA28	Q3ZA28_DEHE1	Putative reductive dehalogenase	*Dehalococcoides mccartyi*
Q3Z6C9	Q3Z6C9_DEHE1	Putative reductive dehalogenase	*Dehalococcoides mccartyi*
Q3Z6C3	Q3Z6C3_DEHE1	Putative reductive dehalogenase	*Dehalococcoides mccartyi*
Q3Z6B3	Q3Z6B3_DEHE1	Putative reductive dehalogenase	*Dehalococcoides mccartyi*
Q3Z693	Q3Z693_DEHE1	Putative reductive dehalogenase	*Dehalococcoides mccartyi*
Q3Z6B6	Q3Z6B6_DEHE1	Putative reductive dehalogenase	*Dehalococcoides mccartyi*
Q0QWE8	Q0QWE8_9CHLR	Trichloroethene reductive dehalogenase	*Dehalococcoides* sp. CH-PCE
Q8GCU2	Q8GCU2_9BACT	Trichloroethene reductive dehalogenase	YK-TCE1
Q8GCU4	Q8GCU4_9CHLR	Trichloroethene reductive dehalogenase	*Dehalococcoides mccartyi*

A0PAT7	A0PAT7_9CHLR	Trichloroethene reductive dehalogenase	uncultured *Dehalococcoides*
Q0QWF8	Q0QWF8_9CHLR	Trichloroethene reductive dehalogenase	*Dehalococcoides*
Q0QWF6	Q0QWF6_9CHLR	Trichloroethene reductive dehalogenase	*Dehalococcoides*
Q5YD55	Q5YD55_DEHSB	Putative chloride reductive dehalogenase	*Dehalococcoides mccartyi*
D2BJ91	D2BJ91_DEHSV	Vinyl chloride reductive dehalogenase	*Dehalococcoides*
D6MZ08	D6MZ08_9CHLR	Putative reductive dehalogenase	*Dehalococcoides*
Q6NAM1	DEHA_RHOPA	Fluoroacetate dehalogenase	*Rhodopseudomonas palustris*
Q1JU72	DEHA_BURSP	Fluoroacetate dehalogenase	*Burkholderia*
P60527	HAD_AGRTR	2-haloalkanoic acid dehalogenase	*Argobacterium tumefaciens*
P72156	ATZA_PSESD	Atrazine chlorohydrolase	*Pseudomonas*
Q01399	DEH2_MORSB	Haloacetate dehalogenase	*Moraxella* sp.
Q01398	DEH1_MORSB	Haloacetate dehalogenase	*Moraxella* sp.
Q93MS3	Q93MS3_9MICC	Halohydrin dehalogenase	*Arthrobacter* sp. AD2
Q8GFE2	CPLR_DESHA	Chlorophenol reductase	*Desulfitobacterium hafniense*

Table S3. Information about the metagenome assemblies.

Sample	Number of contigs	N50	N75	L50	L75	Largest Contig	GC Content (%)
Tanners	131944	1249	737	28718	69125	71886	56.4
Carver	105001	1220	730	23283	55434	75092	53.4
Bennett	18661	970	649	4559	10503	44228	46.6
Turtle	149241	1316	742	29167	75486	83718	56.3
Square	149241	1316	742	29167	75486	83718	56.6
PCB Contaminated Cl-NOM	110839	2435	1030	14074	44525	592351	54.1
PCB Contaminated NOM	129104	2284	997	17025	52943	909738	54.3
Pelican Cl-NOM	37193	2874	1051	3617	13836	144070	53.8
Pelican NOM	92006	2094	963	13201	39266	134506	53.5

qPCR Primer Efficiency

The target efficiency for each primer set was determined by dividing the total number of Illumina sequencing reads by the number of reads that were the correct target gene. More PCR primers were tested but only those with correct target amplification are included. The most common non-target gene amplification was presumed to be primer dimers because no match was made in the NCBI database. The TannersRDase and PCBHaDhg2 primer sets also did also amplify 2-haloacid dehalogenase genes. A target efficiency of greater than 90% was deemed acceptable because this is within the error of qPCR.

Table S4. Efficiency of qPCR primer sets developed in this research, based on amplicon sequencing.

Primer Set Name	Percent Target Efficiency
TannersRDase	90.2
BennettRDase	96
PCBRDase	95.6
PelicanRDase	97.3
TannersHaDhg	90.2
PCBHaDhg1	>99.9
PCBHaDhg2	93.8
CarverHaDhg	91.9
PCB 2-haloacidDhg	>99.9
Pelican 2-haloacidDhg	96.9

qPCR Standards for Novel RDase and Hydrolytic Dehalogenase Genes

>TannersRDase

CACTGCGCGAGTACCGGCGAACCCATCGATTTGCCACACACGCACGCTATCG
GGATACTGGTCGACCAAGACTATCCCACGTCCCACGCCTCGAACGGGCACGA
CTGGATCAGCAACTCCATGAGTTTCATGGCCTATTCGAGCTCAGGGTTCATCG
CCTGCATACTAGCTGACTACATACGCCGGTTGGGCTACTCCGCGCGTGCCCAC
CACGCCCGCAACTATCAAGTCATGGTGCCCCCGATCCTGCTTTGGGCGGGAC
TTGGCGAGATGTGCCGTATCGGCGATACGGTGCTGCACCCCTATCTTGGACCT
CGGTTCAAAGCCGCGGTAGTGACG

>BennettRDase

GTTATTTGATACAGATGCAGGCTCGGTAGACCTCTTCCCTGAGGAGATCGCTT
TTCCAGGGCATTCTTGGGCGCACGCTCCGCAAGATTCGCAAAAGTCCTGAAT
CCCGAGGTCAACAGGCTTATCTGGCTTGAGAGGGAGATCGGTAAACACTTTC
GACAACCTTACCCTTGGGCCAAACTGCTCTGTAATGAGAAGTCCGTGCCTCCC
CAATTCCCCCAGGCCCGCATCGATCGCTAATGGAATACTCAGGGCCGTGTCA
TTTCCCATGGGAATTGCCTTATAACCCAGATTCCTGATAAACTCTGCCATCGT
TACCGCAGTGATGGCCATTTTCGAATAACCCAAACCCGTTGCGGCCGCCGAT
AATGCCCTGGGAGATTCTTTCATCAGTCCATAATCCATCTCAATGGCCATGAC

>PCBRDase

ATCATTCCCATCCTGCTCAATTCTCCAAGACCTGCCTGAACAGCTATGGGAAT
ACTTAATGCAGTGTCATTGCCGCAAGGTATAGCCTGATATCCCAGCTGTCTCA
CAAACGTGGCTACCTTGTGGGCTGTTGCAGCCATGTTGGAGTATTGATTACCG
GCTGCAGCGCTTGCGATTAATGACGGTGCAGTCCGGAAAGCATCATGATTCA
TTTCTAAAGCCAAGGCAATTACACTTTTGGGTTCAAA

>PelicanRDase

GGGGCCGATCGGGTGGGCATCACGCGCCTGAACCCGCTCTGGATCTACACGC
ACTGGGGGATGCAGAACGTCCACTACTCCGGCGCGGCCCAGGCGGGCGACCC
GATCGACATcccccccGAGTACCAGACGGTGATCGTCATGATCCACCGCATGGAT
TACGACGTGATCCTGAGGTCGCCGGCGGTCGAGCATGAAACCGACATCGGGT
ACTCCAAGGCCGCCTGGAGCGCGGCATCGCTCGCCACCTTCATTACTGAACTT
GGGTACAAGGCCATCCCCGCGTGCAACGAGCTCGGCATCAGCATCGCCATGG
CGGTCGATGCCGGCCTTGGCGAAATGGGACGGAACGGCCAGCTG

>TannersHaDhg

TTCAACGGCCTGCCGGGCTTCGCTTTCGCGCCGAACTACCTCGAGTCGCTGCA
CGGCTACGAATGGCTGCGCATGCATTACCTCGACGAGCGCCCCATGGGGACC
GCCAGCGGCCGTACCGTGTTGTGCCTGCACGGCCAGCCAACCTGGTGTTACC
TGTATCGCAAGATGATCCCGGTGTTCCTGGCTGCCGGGCACCGCGTCGTCGC
GCCGGACTTCTTCGGTTTCGGCCGTTCCGACAAACCCGTCGACGACGCGGTCT
ACACGTTCGGTTTCCATCGCGGGATGCTGATGCGCTTCATCGAGACGCTCGAC
CTCCACCGGGTGACG

>PCBHaDhg1

TATGCCGCTAATTCTTCCTCATCCAGATACCGTAGGACACTGTCCGGTAAAAA
ACGTTCAATGAACATGTTCTCTTCCAAGATCAAGCGTTCCCCTGCGGGCGACC
GAAGCGCTTGGAAGACCGGCCGAGCATGTTCGGGCCACATATCCCAACTGAG
TGGTTGGACAATGGCTTCCATATAGACAATAGCCTTGACCCGCTCTGGATGGC
GGTCTGCCCAATGAAAACCCAGCGCCGATCCCCAATCGTGAACCACGAGGGT
GACATTATGTGTTAGCCCCAATGTATCAAACCAGGCATCTAGGTAACGTGCG
TGATCCACGAAACGATAAGTACCATTAGCAATTTTCCCGGAATCTCCCATTCC

CATCAAATCGGGAGCTAAACAACGGCCTACGCCTTCCAAATGGGGAATCACA
TTCCGCCACAGATAGGAGGAAGTGGGATTCCCATGCAAAAAGACAATCGGCT
CCCCCTTTCCTGTATCCACATACGCCAT

>PCBHaDhg2

TGTCACCCTCGTGGTTCACGATTGGGGATCGGCGCTGGGTTTTCATTGGGCAG
ACCGCCATCCAGAGCGGGTCAAGGCTATTGTCTATATGGAAGCCATTGTCCA
ACCACTCAGTTGGGATATGTGGCCCGAACATGCTCGGCCGGTCTTCCAAGCG
CTTCGGTCGCCCGCAGGGGAACGCTTGATCTTGGAAGAGAACATGTTCATTG
AACGTTTTTTACCGGACAGTGTCCTACGGTATCTGGATGAGGAAGAATTAGC
GGCATATCATCGCCCTTACCTTGAACCAGGTGAGGTACGACGTCCCATGCTG
ATGTGGCCGCGCGAACTCCCGTTTGAGGGTGAACCGGGTGATGTGCATGATA
TCGTGGCTCACTATGCGGCTTGGTTGGCGACCAGTACCATTCCCAAGCTTTTC
ATTAACGCCGATCCTGGTTCTATTTTGGTCGGGACACAACGCGAGTTTTGCCG
TACCTGGCCGTGTCAGCAAGAAATCATCG

>CarverHaDhg

TTCGTACTCCTGAGAATCGATTCAAGAATCTACCTCAATATCCCTTTGAACCC
CATTACACCCAGATAGAAGGACTAAGAATGCACTATGTAGATGAGGGACCCA
AGAACGGTGAAGTAGTTTTAATGCTTCATGGTCAGCCAACTTGGTCCTATTTA
TACCGTAAAATGATTCCACCGTTAGCAGTTGCAGGTTATCGAGTGATTGCAGC
TGATCTTATTGGAACGGGTCGATCTGATAAACCCATAGACCTTTCATTTCACA
CTTACGAGTTACATATTCAACGGCTTAAAAAGTTCATTGGTGCTCTGGAACTT
AGAGATGTTACCTTGTTCTGCCAAGACTGGGGTGGTTTGATGGGACTGCGAA
TCGTCGGGGACCAGCCTGATATTTTTGCCAGAGTAGTGGCAGCCAACACAAT
GCTTCCC

>PCB 2-haloacidDhg

AGCACCTCATGCGGCTTTACGCCGAGATTCGATTCAATCAGTTCGTAGGTTCG
CGGCGACGGCTTGAAGGTCTTCGTTGAGTCGATGCTGATGGTCGCGTCGAGA
ATGCTGTCGAGGCCGGTATTGCGCACCAGCGCATTCAGCATGTCGGTGCTGC
CGTTGGAGAGGATCGCGAGCTTGCGGTCTTTCAGCCCAGCAAGCGCCTGTTTC
GCATCCGGGTAAAGATCGAGATGGATGTATTTGTCCATGATGCGATCGAAGA
CGGCTGCGTCGAATGTCAGTCCGAGCGCGTTGAGCGTGAAAGTCAGCGAGTC
GCGTGTGATGACCGAGAAGTCCTCGTAGCGGTTCATTAGCGAGCGCAGCCAG
GTGTACTCAAGCTGCTTCAGCCGCCAGATCTGTGTGATCAGCTCGCCAAAAC
CGGGAAAGGCCTGGTCCGTGACGGCAGCAACTGACTGCACATCGTAGAGTGT
GCCGTAAGCGTCGAATACGACGGCCTTGAT

>Pelican 2-haloacidDhg

ATGATCTTGGGCAGGTCGGCAAGCGCGGAGATTCGAAAGTCGGGTTCCATCC
CGAACTCGTCCATTTGCATGCGCAGCGCCCTGAACATCGACAGCGGGCGCAC
GAGATCGGCTTTCGCAAGCTCGGCAGCCATTGCTTGCGGCGTCACGCGTTCG
ATCCATGCCACATTCAGGCCAAAGGCTTTCGCGCCGCAGGCGTCGAACGGAT

TGGACGACACGAACAGCACCTCATGCGGCTTTACGCCGAGATTCGATTCAAT
CAGTTCGTAGGTTCGCGGCGACGGCTTGAAGGTCTTCGTTGAGTCGATGCTGA
TGGTCGCGTC

2. Additional Results

List of Hydrolytic/Oxidative Dehalogenase Genes Found in Metagenomes

2-haloacid dehalogenase

Haloalkane dehalogenase

Fluoracetate dehalogenase

2,4-dichlorophenol 6-monooxygenase

Pentachlorophenol 4-monooxygenase

2-halobenzoate 1,2-dioxygenase

3-chlorobenzoate-3,4-dioxygenase

Alpha-ketogluterate-dependent 2,4-dichlorophnoxyacetate dioxygenase

Chlorocatechol 2,3-dioxygenase

4-chlorobenzoyl coenzyme A dehalogenase

Atrazine chlorohydrolase

Chlorophenol reductase

Halohydrin dehalogenase

S-triazine hydrolase

Specific Gene Concentrations in Enrichment Cultures

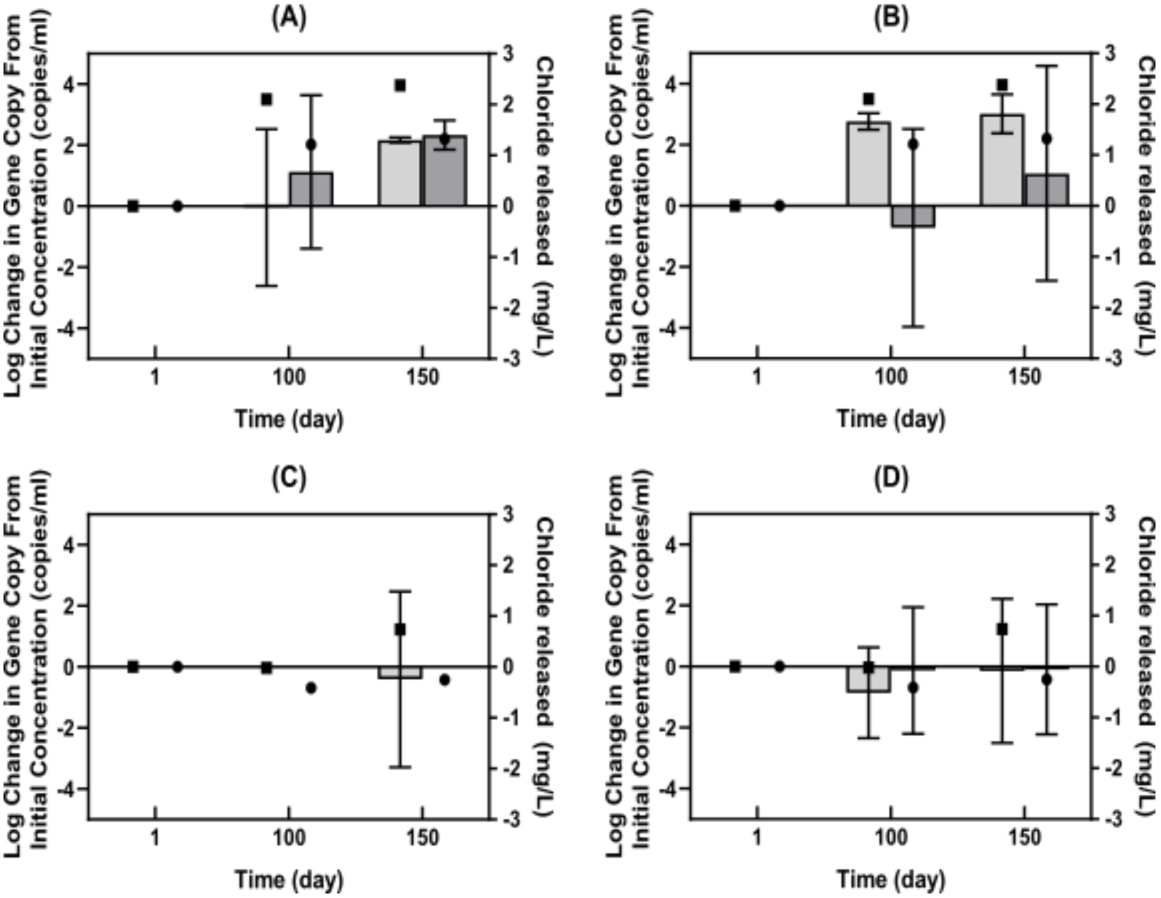

Figure S1. Log change in the gene copy numbers of two RDase genes shown over enrichment time in the Pelican Lake sediment enrichment cultures (A, B) and the PCB soil contaminated soil enrichment cultures (C, D). Shown are the PCBRDase (A, C) and PelicanRDase (B, D). The light grey bars show the Cl-NOM amended treatments and the dark grey bars show the NOM-amended treatments. These genes did not significantly enrich in any of the Cl-NOM-amended enrichments when compared to those amended with NOM. The right y-axis shows the average chloride released at each time point for the Cl-NOM treatments (square) and NOM treatments (circle). One other qPCR primer set targeting a haloalkane dehalogenase gene was developed and tested but this decreased throughout the enrichment period in all replicates and was not detected in the majority of the samples.

Specific Gene Concentrations in Lake Sediments

Table S5. Log gene concentration (copies/g) of reductive dehalogenase genes targeted by qPCR in each of the lake sediments.

	**PCBRDase**	**PelicanRDase**	**BennettRDase**	**TannersRDase**	**Average Chloride**	**Percent Road Density**
Big Marine	Below LOQ	2.86	0.00	1.58	11	<18
Tanners	1.08	1.75	Below LOQ	3.77	161.7	29
White Bear	1.30	1.27	Below LOQ	3.13	35.3	<18
Gervais	Below LOQ	Below LOQ	Below LOQ	Below LOQ	150	29
Carver	Below LOQ	Below LOQ	0.00	1.43	190	25
Bennett	0.00	2.43	2.34	0.00	160	23
Square	Below LOQ	1.28	Below LOQ	2.23	7	<18
Long	Below LOQ	Below LOQ	0.00	Below LOQ	151	33
Turtle	Below LOQ	Below LOQ	Below LOQ	0.00	40	<18
Wakefield	Below LOQ	3.87	Below LOQ	3.55	146	26
Johanna	1.50	1.23	0.00	Below LOQ	153	36
Centerville	Below LOQ	Below LOQ	0.00	0.00	26	<18
Como	1.01	3.10	Below LOQ	2.73	193	32
Josephine	1.00	1.92	Below LOQ	1.56	65	21

Table S6. Log gene concentrations (copies/g) of the hydrolytic/oxidative dehalogenase genes targeted by qPCR in each of the lake sediments.

	**PCB HAdhg**	**PCB 2haloacid**	**PCB HaDhg**	**Carver HaDhg**	**Tanners HaDhg**	**Pel S2haloacid**	**Average Chloride**	**Percent Road Density**
Big Marine	Below LOQ	1.01	0.00	2.94	2.29	0.00	11	<18
Tanners	1.00	2.05	Below LOQ	3.47	2.89	Below LOQ	161.7	29
White Bear	1.04	Below LOQ	Below LOQ	3.30	4.29	1.04	35.3	<18
Gervais	Below LOQ	Below LOQ	0.00	1.80	1.44	Below LOQ	150	29
Carver	Below LOQ	Below LOQ	Below LOQ	2.19	2.41	Below LOQ	190	25
Bennett	0.00	1.34	0.00	2.67	2.80	0.00	160	23
Square	Below LOQ	Below LOQ	0.00	3.06	2.21	Below LOQ	7	<18
Long	Below LOQ	Below LOQ	Below LOQ	1.10	1.03	Below LOQ	151	33
Turtle	Below LOQ	Below LOQ	0.00	1.89	1.57	Below LOQ	40	<18
Wakefield	1.20	1.79	Below LOQ	3.07	3.04	1.03	146	26
Johanna	1.83	Below LOQ	Below LOQ	2.30	1.45	1.27	153	36

Centerville	1.00	Below LOQ	0.00	2.03	1.66	Below LOQ	26	<18
Como	Below LOQ	1.98	Below LOQ	3.51	2.55	Below LOQ	193	32
Josephine	Below LOQ	1.19	Below LOQ	2.90	2.26	0.00	65	21

Table S7. Variations in RDase and hydrolytic/oxidative dehalogenase genes over time (April, 2016 to March, 2017). Numbers are listed as the log of the gene concentration (copies/ml) for each of the sampling dates. Genes that were detected but below the limit of quantification are denoted as below LOQ. A value of 0 is no detection.

**Lake**	**Gene**	**4/7/2016**	**5/30/2016**	**7/24/2016**	**9/11/2016**	**11/20/2016**	**3/25/2017**	**Average**
Tanners	PCBHaDhg1	Below LOQ	Below LOQ	1.02	1.0	Below LOQ	Below LOQ	0.55
	PCBRDase	Below LOQ	Below LOQ	Below LOQ	Below LOQ	0	Below LOQ	Below LOQ
	TannersRDase	2.58	2.56	2.36	2.51	Bql	2.58	2.21
	CarverHaDhg	3.30	3.02	3.00	3.23	1.10	3.43	2.85
	PelicanRDase	Below LOQ	Below LOQ	Below LOQ	Below LOQ	0	Below LOQ	Below LOQ
	BennettRDase	Below LOQ	Below LOQ	Below LOQ	Below LOQ	Below LOQ	Below LOQ	Below LOQ
	TannersHaDhg	2.83	2.68	2.65	2.85	1.36	3.01	2.56
	PCB 2-haloacidDhg	2.20	1.96	1.86	2.23	Below LOQ	2.09	1.79
	PCBHaDhg2	Below LOQ	Below LOQ	Below LOQ	Below LOQ	0	Below LOQ	Below LOQ
	Total 16S rRNA Gene	8.76	7.43	8.03	7.94	5.56	8.10	7.55
Bennett	PCBHaDhg1	1.10	Below LOQ	Below LOQ	Below LOQ	Below LOQ	Below LOQ	Below LOQ

Lake	Gene	4/7/2016	5/30/2016	7/24/2016	9/11/2016	11/20/2016	3/25/2017	Average
	PCBRDase	0	0	Below LOQ	Below LOQ	0	0	0
	TannersRDase	0	0	0	0	0	0	0
	CarverHaDhg	3.01	3.30	2.81	2.02	1.65	3.25	2.67
	PelicanRDase	2.64	3.33	2.79	1.37	1.71	2.73	2.43
	BennettRDase	1.07	2.70	2.41	2.82	1.97	3.07	2.34
	TannersHaDhg	3.91	3.06	2.72	2.29	1.67	3.13	2.80
	PCB 2-haloacidDhg	1.59	1.75	1.32	1.21	1.05	1.08	1.34
	PCBHaDhg2	0	0.30	0.52	Below LOQ	0.00	Below LOQ	Below LOQ
	Total 16S rRNA Gene	7.49	8.09	8.26	7.7	7.24	7.79	7.76
Carver	PCBHaDhg1	Below LOQ	Below LOQ	Below LOQ	Below LOQ	0	Below LOQ	Below LOQ
	PCBRDase	0	0	Below LOQ	0	0	0	Below LOQ
	TannersRDase	Below LOQ	1.13	1.42	1.53	0	Below LOQ	1.02
	CarverHaDhg	3.21	3.06	3.74	3.47	2.40	3.41	3.22

Lake	Gene	4/7/2016	5/30/2016	7/24/2016	9/11/2016	11/20/2016	3/25/2017	Average
	PelicanRDase	Below LOQ	Below LOQ	1.15	Below LOQ	0	Below LOQ	0.50
	BennettRDase	0	Below LOQ	0	4.72	Below LOQ	Below LOQ	0.97
	TannersHaDhg	2.46	2.25	3.40	3.64	1.88	2.34	2.66
	PCB 2-haloacidDhg	Below LOQ	1.31	1.49	1.57	Below LOQ	1.17	1.18
	PCBHaDhg2	0	0	0	0	0	0	0
	Total 16S rRNA Gene	7.27	7.38	8.18	7.66	6.41	7.33	7.38
Turtle	PCBHaDhg1	Below LOQ	Below LOQ	Below LOQ	Below LOQ	0	Below LOQ	0.30
	PCBRDase	0	0	0	0	0	0	Below LOQ
	TannersRDase	0	1.12	0	0	0	0	0.19
	CarverHaDhg	2.25	2.19	2.77	2.33	1.53	2.37	2.24
	PelicanRDase	Below LOQ	Below LOQ	Below LOQ	0	0	Below LOQ	0.13
	BennettRDase	0	Below LOQ	0	Below LOQ	0	Below LOQ	-0.24
	TannersHaDhg	1.14	Below LOQ	1.62	1.39	Below LOQ	1.26	1.17

Lake	Gene	4/7/2016	5/30/2016	7/24/2016	9/11/2016	11/20/2016	3/25/2017	Average
	PCB 2-haloacidDhg	Below LOQ	Below LOQ	Below LOQ	Below LOQ	Below LOQ	Below LOQ	Below LOQ
	PCBHaDhg2	0	0	0	0	0	0	0
	Total 16S rRNA Gene	7.35	7.41	7.64	7.16	6.57	7.38	7.22
Square	PCBHaDhg1	0	Below LOQ	Below LOQ	Below LOQ	0.00	Below LOQ	Below LOQ
	PCBRDase	0	0	0	0	0	Below LOQ	Below LOQ
	TannersRDase	Below LOQ	Below LOQ	0	Below LOQ	0.00	0	Below LOQ
	CarverHaDhg	2.15	1.53	1.75	2.38	Below LOQ	2.50	1.79
	PelicanRDase	0	0.00	0.00	0	0.00	Below LOQ	Below LOQ
	BennettRDase	0	Below LOQ	Below LOQ	0	Below LOQ	Below LOQ	Below LOQ
	TannersHaDhg	2.28	2.34	2.49	2.60	Below LOQ	3.20	2.31
	PCB 2-haloacidDhg	Below LOQ	Below LOQ	0.00	1.20	0	0.88	0.25
	PCBHaDhg2	0.00	0.00	Below LOQ	0.00	0.00	0.00	Below LOQ
	Total 16S rRNA Gene	8.14	7.18	6.87	7.17	5.73	7.66	7.02

Correlations of Functional Genes with Chloride

Table S8. Statistical comparison of the RDase and hydrolytic/oxidative dehalogenase genes to chloride impairment (point biserial correlation) or chloride concentration (Spearman's Rank Correlation).

	**Point Biserial Correlation**		**Spearman's Rank Correlation**	
**Gene**	***P*-value**	$\mathbf{r_{pb}}$	***P*-value**	**ρ**
PCBRDase	0.43	0.05	0.82	0.07
PCBHaDhg1	0.16	0.28	0.89	-0.04
PelicanRDase	0.31	0.15	0.69	0.12
PCB 2-haloacidDhg	0.11	0.35	0.27	0.32
BennettRDase	0.24	0.20	0.40	0.25
PCBHaDhg2	Below LOQ		Below LOQ	
TannersRDase	0.43	0.05	0.98	0.01
CarverHaDhg	0.34	-0.12	0.81	0.07
TannersHaDhg	0.36	-0.11	0.60	0.15
Pelican 2-haloacidDhg	0.41	0.07	0.86	0.05

Table S9. Concentrations of several target organohalide respiring bacteria in the lake sediments (copies/g). Samples that were detected but below the limit of quantification are denoted as Below LOQ.

**Copies/g wet weight**	***Geobacter***	***Desulfomonile***	***Dehalobium***	***Anaeromyxobacter***	***Sulfurospirillum***	***Desulfovibrio***
Big Marine	1.3×10^{7}	3.3×10^{3}	8.8×10^{1}	3.7×10^{5}	2.3×10^{1}	2.5×10^{3}
Tanners	4.5×10^{7}	4.1×10^{3}	1.9×10^{2}	2.2×10^{5}	3.4×10^{2}	5.9×10^{3}
White Bear	2.7×10^{7}	7.9×10^{3}	1.2×10^{2}	1.2×10^{5}	9.1×10^{2}	9.0×10^{3}
Gervais	2.9×10^{6}	8.3×10^{2}	3.6×10^{1}	7.8×10^{4}	1.7×10^{1}	1.6×10^{3}
Carver	5.2×10^{6}	1.4×10^{3}	3.3×10^{2}	5.8×10^{4}	1.0×10^{1}	2.0×10^{3}
Bennett	4.8×10^{6}	2.2×10^{3}	1.5×10^{2}	8.5×10^{4}	9.1×10^{1}	1.9×10^{3}
Square	6.5×10^{6}	2.7×10^{3}	6.0×10^{1}	2.8×10^{5}	1.0×10^{0}	8.8×10^{3}
Long	6.8×10^{6}	3.8×10^{3}	2.7×10^{2}	3.5×10^{5}	2.4×10^{1}	6.9×10^{3}
Turtle	4.9×10^{6}	8.9×10^{3}	1.6×10^{2}	2.1×10^{5}	Below LOQ	3.7×10^{3}
Wakefield	1.1×10^{8}	3.7×10^{3}	2.4×10^{2}	2.4×10^{5}	5.0×10^{2}	4.2×10^{3}
Johanna	1.7×10^{7}	1.3×10^{2}	1.1×10^{1}	4.2×10^{5}	3.3×10^{2}	5.0×10^{3}
Centerville	3.3×10^{6}	3.0×10^{2}	1.6×10^{2}	8.8×10^{4}	2.1×10^{8}	1.3×10^{3}

**Copies/g wet weight**	***Dehalogenimonas***	***Dehalococcoidia***	***D. mccartyi***	***Dehalobacter***	***Desulfitobacterium***	**Total 16S**
Como	3.4×10^{8}	2.1×10^{4}	2.4×10^{2}	1.1×10^{6}	2.2×10^{3}	7.3×10^{3}
Josephine	2.2×10^{7}	1.5×10^{3}	8.7×10^{1}	6.5×10^{5}	9.7×10^{1}	2.8×10^{3}
Big Marine	6.5×10^{4}	8.0×10^{6}	3.6×10^{3}	5.9×10^{2}	6.3×10^{1}	4.65×10^{7}
Tanners	2.7×10^{5}	1.8×10^{7}	1.1×10^{4}	1.9×10^{3}	6.5×10^{1}	1.5×10^{7}
White Bear	4.0×10^{4}	1.7×10^{7}	7.1×10^{3}	8.8×10^{2}	3.9×10^{2}	3.9×10^{6}
Gervais	3.2×10^{4}	4.3×10^{6}	5.7×10^{3}	4.6×10^{2}	2.6×10^{1}	6.8×10^{7}
Carver	9.7×10^{4}	8.0×10^{7}	9.2×10^{3}	2.2×10^{2}	3.1×10^{1}	3.1×10^{7}
Bennett	5.0×10^{4}	1.7×10^{7}	1.0×10^{4}	3.5×10^{3}	3.7×10^{1}	2.2×10^{7}
Square	1.8×10^{5}	4.2×10^{6}	7.5×10^{3}	2.6×10^{2}	3.3×10^{2}	7.6×10^{7}
Long	1.5×10^{5}	1.9×10^{7}	1.1×10^{4}	2.0×10^{3}	7.4×10^{1}	7.6×10^{7}
Turtle	1.3×10^{5}	1.3×10^{7}	2.9×10^{3}	6.3×10^{2}	5.3×10^{1}	4.6×10^{7}
Wakefield	1.4×10^{5}	1.4×10^{7}	4.0×10^{4}	1.4×10^{3}	1.1×10^{2}	3.6×10^{7}
Johanna	1.9×10^{4}	5.8×10^{5}	7.7×10^{3}	1.4×10^{3}	1.0×10^{2}	1.1×10^{7}
Centerville	4.9×10^{4}	1.1×10^{6}	1.8×10^{3}	Below LOQ	2.3×10^{1}	3.7×10^{7}
Como	3.1×10^{5}	3.5×10^{7}	2.2×10^{4}	2.3×10^{3}	2.0×10^{2}	8.9×10^{6}

Josephine	9.7×10^4	7.3×10^6	2.5×10^3	5.1×10^2	5.0×10^1	3.7×10^7

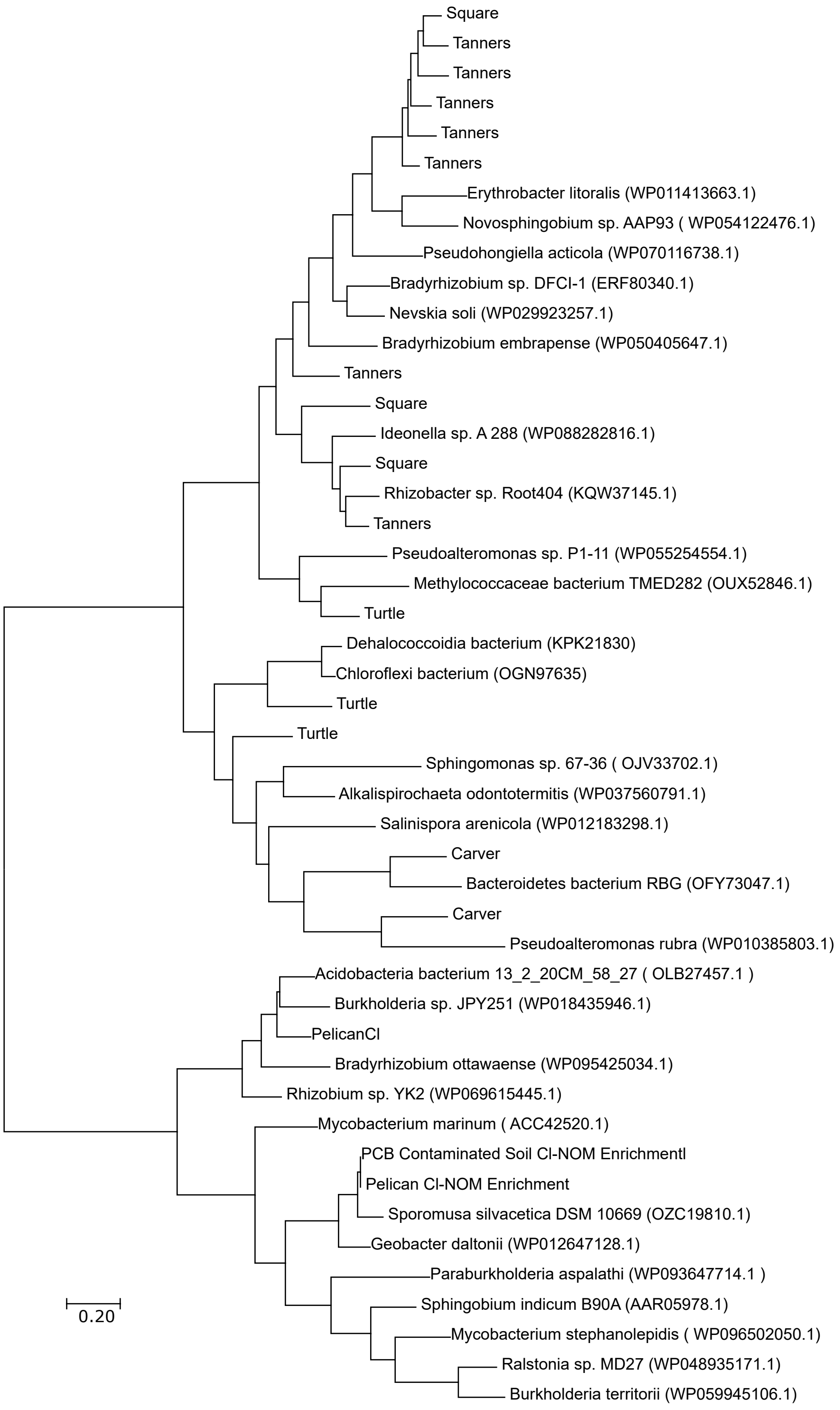

Figure S2. Taxonomic relationships of the putative haloalkane dehalogenase genes sequenced in the urban lakes and the Cl-NOM enrichment cultures. The identified genes were compared to haloalkane dehalogenase genes in the NCBI database. A reductive dehalogenase gene was used as an outgroup. The tree was constructed using the maximum likelihood algorithm in Mega5. Sequences were aligned using Clustal.
